# Morphological and Molecular Characterization of Toxigenic *Aspergillus flavus* from Groundnut Kernels in Kenya

**DOI:** 10.1155/2020/8854718

**Published:** 2020-09-07

**Authors:** Robert O. Okayo, Darius O. Andika, Mathews M. Dida, George O. K'Otuto, Bernard M. Gichimu

**Affiliations:** ^1^Department of Plant, Animal and Food Sciences, Jaramogi Oginga Odinga University of Science and Technology, Bondo, Kenya; ^2^Department of Applied Sciences, Maseno University, Maseno, Kenya; ^3^Department of Agricultural Resource Management, University of Embu, Embu, Kenya

## Abstract

Pathogenesis of *Aspergillus flavus* on important agricultural products is a key concern on human health due to the synthesis and secretion of the hazardous secondary metabolite, aflatoxin. This study identified and further characterized aflatoxigenic *A. flavus* from groundnuts sampled from sundry shops in Kenya using integrated morphological and molecular approaches. The groundnuts were plated on potato dextrose agar for isolation and morphological observation of *A. flavus* based on macroscopic and microscopic features. Molecular characterization was done through amplification and comparison of the partial sequence of the ITS1-5.8S-ITS2 region. The expression analysis of *aflR*, *aflS*, *aflD*, *aflP,* and *aflQ* genes in the aflatoxin biosynthesis pathways was conducted to confirm the positive identification of *A. flavus*. The gene expression also aided to delineate toxigenic isolates of *A. flavus* from atoxigenic ones. Morphologically, 18 isolates suspected to be *A. flavus* were identified. Out of these, 14 isolates successfully amplified the 500 bp ITS region of *A. flavus* or *Aspergillus oryzae,* while 4 isolates were not amplified. All the remaining 14 isolates expressed at least one of the aflatoxigenic genes but only 5 had all the genes expressed. Partial sequencing revealed that isolates 5, 11, 12, 13, and 15 had 99.2%, 97.6%, 98.4%, 97.5%, and 100% homology, respectively, to the *A. flavus* isolate LUOHE, ITS-5.8S-ITS2, obtained from the NCBI database. The five isolates were accurate identification of atoxigenic *A. flavus*. Precise identification of toxigenic strains of *A. flavus* will be useful in establishing control strategies of the fungus in food products.

## 1. Introduction

*Aspergillus flavus* is a facultative pathogen capable of existing in diverse ecological niches [1]. It survives optimally in the tropics at relatively high temperature of about 28°C to 37°C and high relative humidity of about 95% [2]. It derives its energy as a saprophyte on plant debris rich in carbohydrates [3]. Like other fungi, *A. flavus* does synthesize and release a plethora of secondary metabolites such as aflatoxin B1 and aflatoxin B2, aspergillic acid, aflam, nitropropionic acid, and kojic acid [4]. These metabolites act as virulence factors during pathogenicity, as communication signals, and for ecological adjustments to suit their existence [5].

Colonization of *A. flavus* and the succeeding aflatoxin contamination have been found in agriculturally important crops like maize, legumes, nuts, and their products [6]. In Kenya, the outbreak of aflatoxin contamination in the years 2004 and 2005 focused most of the aflatoxin research efforts on maize [7, 8]. This is because maize is a staple food and contributes majorly to the daily diet of most households in the country. However, the reported high incidences of *A. flavus* on groundnuts (*Arachis hypogea*) in Western Kenya [9] underscore the need for greater vigilance and surveillance of other foodstuffs other than maize. The current study is one such effort targeting groundnut which is a nonstaple foodstuff that is a delicacy to many Kenyan communities especially in Western Kenya region.

Exposure to low dosage of aflatoxin overtime leads to chronic aflatoxicosis that leads to poor feed conversion and stunting growth in children, immune suppression, and reduction in life expectancy [10]. Exposure to high aflatoxin doses that are greater than 1000 ppb leads to acute aflatoxicosis, within a relatively short time, which is characterized by liver damage, hepatitis, and, in some cases, death [11]. The International Agency for Research on Cancer (IARC) documented aflatoxin B1 as the most lethal and classified it as probable human carcinogen [12]. However, only 40–50% of *A. flavus* strains can produce the toxins [13] and hence there is need to distinguish the toxic from the nontoxic ones.

Morphological classification is one of the ancient means of distinguishing the species in *Aspergillus* section *Flavi,* though it lacks precision owing to close resemblance of these species [14]. It is, however, necessary in aiding the grouping of *Aspergillus* isolates into sections which allows easier scrutiny using advanced characterization methods such as molecular and biochemical tools [15]. Morphological classification employs the use of numerous available taxonomical keys for *Aspergillus* species identification at macroscopic and microscopic levels [16]. The keys delineated features such as conidia, conidiophore, mycelial colour, colony reverse colour, colony diameter, exudates production, and the sclerotia and cleistothecia formation for macroscopic characterization [17, 18]. The microscopic characterization depends on vesicles shape, size and seriation, stipe, hülle cells formation, and cleistothecium [17–19].

Morphological similarities at both interspecific and intraspecific levels within the *Aspergillus* genera hamper the use of the morphology-based taxonomical keys for distinguishing the various species [19, 20], thus leading to inaccurate identification. In addition, the morphological methods are laborious, are time-consuming and require skilled microbiologists [19]. A complex mycobiota contains numerous compounds and possibly species that may hinder sensitivity and efficiency of detection of specific species. Molecular approach involving amplification of the variable regions of the known DNA target followed by sequencing is an appropriate molecular approach for identification of closely related members of the Aspergilli [15, 21]. However, morphological characterization is instrumental in categorizing the isolates into groups or sections that may thereafter be cultured for easier and specific diagnosis in pure culture by other methods.

Molecular tools such as polymerase chain reaction (PCR) based methods that target and amplify the specific DNA regions for specific fungal species are better alternatives for sensitive and rapid diagnosis [22, 23]. The most commonly targeted genome regions for identification of *Aspergillus* species are the highly variable sequence of the internal transcribed spacer (ITS) regions and intergenic spacer (IGS) of the ribosomal DNA (rDNA) and nuclear genes, RNA polymerase II (*rbp2*), and *β*-tubulin (*benA*) [24, 25]. To a lesser extent, two mitochondria genes, small rRNA subunit (*rns*) and cytochrome oxidase subunit 1 (*cox1*), are also used for the identification and phylogenetic studies [26]. The species *A. oryzae* is believed to be the domesticated form of aflatoxigenic *A. flavus* and is known to have its aflatoxin biosynthetic genes silent [5, 27]. Therefore, investigating the aflatoxin biosynthetic genes expression could provide an additional separation mechanism not only for *A. oryzae* from *A. flavus* but also to separate toxigenic strains of *A. flavus* from atoxigenic ones.

The aflatoxin biosynthesis pathways consist of approximately 30 genes and 27 enzymatic steps [13, 28]. They consist of structural and regulatory genes [29]. The most essential structural genes that encode for the key enzymes in the aflatoxin production are *aflD, aflO, aflQ, aflM,* and *aflP* [29]. According to Sweeney et al. [22], the expression patterns of these structural genes are positively correlated to the aflatoxin production capacity. The *aflR* and *aflS* genes found in the middle of the gene clusters regulate the expression of the structural genes [30, 31]. The *aflR* genes are well characterized and shown to encode the 47 kDa sequence-specific zinc finger DNA binding proteins, while the *aflS* is less characterized but its expression shows correlation with the aflatoxin production capacity [30].

This study sought to identify and characterize aflatoxigenic *A. flavus* from groundnuts using an integration of morphological and molecular approaches. Correct identification of the toxigenic *A. flavus* is important given that only 40–50% of *A. flavus* strains can produce the toxins [13] and are thus harmful to the consumer. In addition, groundnut host specific toxin producing *A. flavus* strains provide great tools for use in screening groundnut varieties for resistance/tolerance to *A. flavus*. We believe that this will form the first report in Kenya to characterize *A. flavus* from groundnuts using a combination of morphological and molecular approaches.

## 2. Materials and Methods

### 2.1. Groundnut Sample Collection

Groundnut samples were obtained from sundry shops in Bondo town in Bondo Sub-County, Siaya County, Kenya. The shops were selected randomly. On each sack of seeds, the samples were picked from the bottom, middle, and top. A composite sample was then obtained from the seeds drawn and 100 seeds from the composite were placed on a well labelled paper bag. The samples were kept at a room temperature in biology laboratory at Jaramogi Oginga Odinga University of Science and Technology (JOOUST), before fungal isolation.

### 2.2. Isolation of *Aspergillus flavus* Isolates

**Figure 1:**
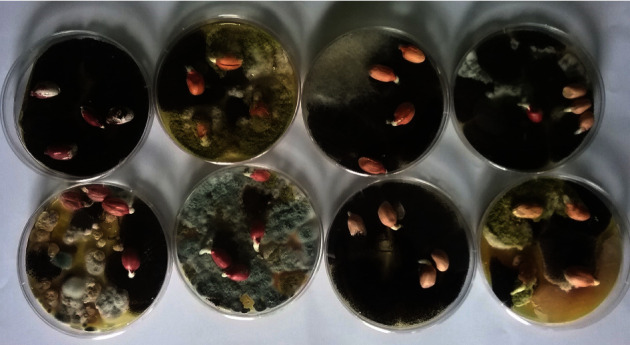
Complex microbiota growth on groundnut kernels incubated on PDA media.

The seeds were surface sterilized in accordance with the method of Samson et al. [32]. Seeds from the sundry shops were thoroughly mixed after which approximately 100 seeds were randomly selected and washed in 350 ml of 0.5% ethanol solution and rinsed with distilled water twice. Four kernels were randomly obtained and plated using sterile forceps onto PDA (20 g dextrose, 4 g Potato extract, and 15 g Agar) growth media and incubated at 28°C for 10 days (Figure 1). Any visible *A. flavus*-like mycelial growth or spores characterized by greenish colouration was considered as the initial isolation criterion.

### 2.3. Morphological Characterization of *Aspergillus flavus* Isolates

Macroscopic features of the isolates including colony growth, colour, texture, conidia, and reverse colour were observed after 10 days of inoculation [17, 32]. For microscopic assessment, the slide culture was prepared according to Diba et al. [33]. The 18 × 18 mm cover slip was placed gently at an angle of 45° on inoculated culture agar media. Upon fungus sporulation, the cover slip was gently removed and placed on the microscope slide and a drop of lacto-fuchsine was added and covered with a small cover slip. Another drop of lactofuchsin was placed on top of the small cover slip before completing the assembly with a 22 × 22 mm cover slip. The microscopic features such as conidiophores, vesicles, metulae, philiades, conidia shape, and texture were observed under a miotic BA 210 basic biological light microscope using the immersion oil (100x) objective.

### 2.4. DNA Extraction

The conidia of isolates representative of *A. flavus* was taken from a 10-day-old culture on a PDA media and inoculated in a 150 ml potato dextrose broth (PDB) in 250 ml conical flask. The broth was incubated under agitation at 120 rpm for 72 hours at 30°C. The Whatman filter paper No. 1 was used to harvest the mycelia through filtration. The harvested mycelial mats were freeze dried for 48 hours and kept at deep freezer at −80°C. Extraction of genomic DNA was done following the method of Diniz et al. [34] with slight modifications. One gram of fresh mycelia was put in precooled motor and ground into a fine powder. Lysis buffer (1.5 ml) was added to the mycelia powder and incubated at 69°C for 20 minutes in a shaker. Using a temperature-controlled centrifuge, the suspension was centrifuged at 13000 rpm for 15 minutes at 4°C. The supernatant was then transferred into a new tube and 0.75 ml of 4 M sodium acetate was added to precipitate polysaccharides and proteins at a pH of 5.2. The solution was mixed thoroughly by inversion and incubated in ice for 20 minutes. The resulting solution was centrifuged at 12000 rpm for 20 minutes and the resulting supernatant was transferred into a new tube, where 0.175 ml isopropanol was added and mixed gently by inversion and placed in ice for 15 minutes. The DNA was pelletized by centrifugation at 13000 rpm for 15 minutes at 4°C. The pellets were washed with 70% ethanol and air dried by letting centrifuge tube containing the pallet to sit upside down on a paper towel to dry. After drying, 0.1 ml of TE buffer at a pH 8 was added and spinned briefly for 10 minutes. The DNA was stored in a refrigerator at −20°C.

### 2.5. Amplification of Internal Transcribed Spacer Region

The primers FLA1 (5′-GTAGGGTTCCTAGCGAGCC-3′) and FLA2 (5′-GGAAAAAGATTGATTTGCGTTC-3′) designed on the basis of sequence alignments [24] were used to amplify the partial sequence of ITS region of the rDNA. The PCR reaction was done in 25 *μ*l PCR tubes containing 2.5 *μ*l of the DNA template, 1.5 *μ*l of each primer set, 2.5 *μ*l of reaction buffers, 1 *μ*l of MgCl_2_, 0.25 *μ*l of dNTPs, and 0.2 *μ*l of Taq DNA polymerase. The PCR amplification parameters were set as follows: initial denaturation cycle of 5 minutes at 95°C, 30 cycles of 30 seconds each at 95°C for the subsequent denaturation, 30 cycles of 30 seconds each at 58°C for annealing, 30 cycles of 45 seconds at 72°C for extension, and final cycle of 5 minutes at 72°C for final extension. The PCR products were then held at 4°C indefinitely and visualized in 1.2% agarose gels in TE buffer and compared with 100 bp DNA molecular ladder. Electrophoresis was conducted at 80 V for 1 hour and the gel was observed under UV light. A single band of approximately 500 bp was expected when the amplified ITS was from *A*. *flavus*.

### 2.6. PCR Purification and Sequencing

The gel bands were cut with the aid of blue light and purified using the quick protocol for DNA gel purification by vacuum (wizard® SV Gel and PCR clean-up system). The sliced DNA band was placed into a microcentrifuge tube and 10 *μ*l of membrane binding solution added, vortexed, and incubated at 65°C in water bath until the gel dissolves. The solution was transferred into a minicolumn inserted into a microcentrifuge tube, allowed to incubate at room temperature on the bench for 3 minutes and centrifuged at 1600 rpm for 5 minutes. 700 *μ*l of the membrane wash solution was added and centrifuged at 1600 rpm for 5 minutes and the flow through discarded. Two additional washing steps were done with 500 *μ*l of membrane wash solution. The column assembly lid was opened to allow the residual ethanol to evaporate on the bench. Nuclease-free water was used to elute the DNA on the minicolumn, incubated for 1 minute, and then centrifuged for 2 minutes. The purified DNA was sequenced at Intertek Laboratory in Australia. The obtained sequences were aligned with the sequence of *A. flavus* isolate LUOHE (Accession no. MT645222.1) obtained from the NCBI GenBank using Clustal X 2.1 software (http://www.clustal.org/clustal2/).

### 2.7. Analysis of Aflatoxin Biosynthetic Gene Expression

#### 2.7.1. Growth Condition

The isolates were grown on a PDA at 20°C until sporulation and inoculum of conidia suspension were harvested with 0.1% Tween 80 (v/v). The isolates were each grown in aflatoxin inducing Yeast Extract Sucrose (YES) (yeast-extract-sucrose: 2% yeast extract, 15% sucrose) media and aflatoxin-repressing Yeast Extract Peptone (YEP) (yeast-extract peptone: 2% yeast extract, 15% peptone) media broth in 500 ml flask. The cultures were incubated in the dark for 7 days until enough mycelia growth was observed and then harvested.

#### 2.7.2. RNA Extraction

The harvested mycelia were ground into a fine powder using a cooled pestle and mortar and 100 mg of tissue transferred into a 10 ml centrifuge tube. The cells were lysed through addition of 5 ml of lysis buffer. The mixture was then vortexed and incubated at 65°C in a water bath for 20 minutes with gentle whirling and then allowed to cool for 20 minutes on the bench. Two (2) ml of chloroform was added and the mixture was thoroughly vortexed and incubated in ice for 5 minutes before centrifuging at 8,000 rpm for 20 minutes at 4°C. The clear supernatant was gently moved to a new RNase free tube and 1.5 ml of lithium chloride (LiCl) was added and mixed by inversion and incubated at −80°C for 2 hours. The solution was then centrifuged at 8,000 rpm for 20 minutes in a refrigerated centrifuge to pellet the RNA. The pellets were washed with 75% ethanol and centrifuged at 10,000 rpm for 10 minutes at 4°C. The pellets were then air dried and dissolved in 100 *μ*L of RNase free water. The concentration and quality of RNA were assessed with NanoDrop 1000 Spectrophotometer.

#### 2.7.3. Reverse Transcriptase PCR

Reverse transcriptase PCR (RT-PCR) analysis was used to detect expression of aflatoxin biosynthetic genes. This was done according to Superscript TM Reverse transcriptase kit. The reverse transcription was done in 20 *μ*l nuclease-free microcentrifuge tube containing 1 *μ*l of primer pairs, 5 *μ*l of isolated RNA, 1 *μ*l of each dNTP mix at a neutral pH (dATP, dGTP dCTP, and dTTP), and 13 *μ*l of distilled water. The mix was then incubated at 65°C for 5 minutes and incubated on ice for 5 minutes. Brief centrifugation was done to collect the mix at the bottom and 4 *μ*l of the first strand buffer, 1 *μ*l of RNase inhibitor, 1 *μ*l of DTT, and 1 *μ*l of superscript were added and mixed through pipetting up and down. The incubation was then done for 45 minutes at 55°C. The reaction was stopped by heating at 70°C for 15 minutes in a water bath. The RT-PCR was done according to Rodrigues et al. [35]: 1 cycle of 4 minutes for initial denaturation at 94°C, 30 cycles of 60 seconds for subsequent denaturation at 94°C, 30 cycles of 1 minute for annealing at 55–60°C, and 30 cycles of 1 minute for extension at 72°C and a final extension at 72°C for 6 minutes.

#### 2.7.4. Reverse Transcription-PCR Primers

The RT-PCR primers (Table 1) were designed using the primer 3 plus software and the expression of regulatory genes *aflR* and *aflS* and three structural genes, *aflQ, aflP,* and *aflD,* were analysed. This was done to distinguish isolates with the potential of aflatoxin production from nonproducers. The *tub1* gene encoding for *β*-tubulin was used as a housekeeping gene.

## 3. Results

### 3.1. Macroscopic Morphological Features

Microbiota growth was observed on the PDA media (Figure 1). Only the greenish coloured spores were needle picked and transferred onto new petri dishes with fresh PDA media for purification. Approximately more than 200 isolates based on the greenish coloration were picked and inoculated on a fresh media.

**Figure 2:**
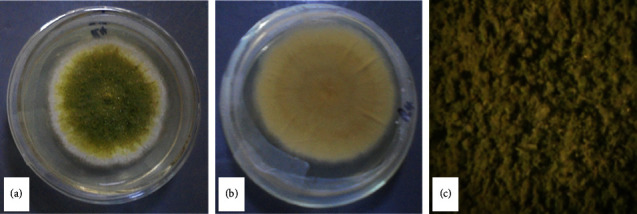
Macroscopic features of *Aspergillus flavus* isolate 5 colony showing the greenish conidia encircled with a white border (a); the reverse cream colour of the colony (b); and the colony texture (c).

Sporulation began after five days from the centre and progressed radially covering the surface of the colony. The conidia produced had yellowish to olive colour. As the sporulation spread outwards, it gave a characteristic white border encircling the sporulating mycelia (Figure 2(a)). The white border was eventually covered as the entire mycelia continued to sporulate and to produce more conidia by day 10. These colonies had clear exudates and cream colour on the reverse (Figure 2(b)). The isolates representative of *A. flavus* had a greenish colony that spread radially from the point of inoculation. As the colony progressively grew, it become slightly raised as mycelia piled and the centre becomes floccose and rough (Figure 2(c)). Eighteen uncontaminated isolates were obtained.

### 3.2. Microscopic Morphological Features

**Figure 3:**
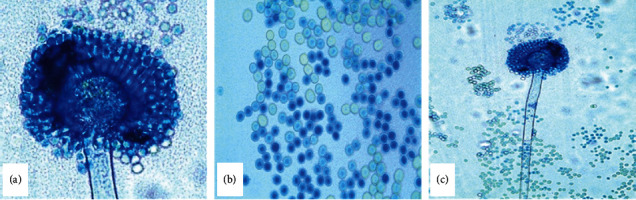
The microscopic characteristics of *Aspergillus flavus* isolate 5 under the basic biological light microscope showing the biseriate with philiades radiating from all sides (a); the globose conidia with varying sizes that are slightly roughened (b); and unbranched conidiophore which is nonseptate, rough, and hyaline (c).

The microscopic features of *A. flavus* showed that the colonies were biseriate with philiades radiating in all sides from metulae that were borne on subglobose or globose vesicles of variable size. The metulae obscured the entire surface of the vesicles (Figure 3(a)). The conidia had a globose shape ranging between 250 *μ*m and 450 *μ*m in diameter with thin walls and rough texture (Figure 3(b)). The conidiophores had a rough texture and thick walls were nonpigmented and unbranched (Figure 3(c)).

### 3.3. Detection of *Aspergillus flavus* Using PCR Method

**Figure 4:**
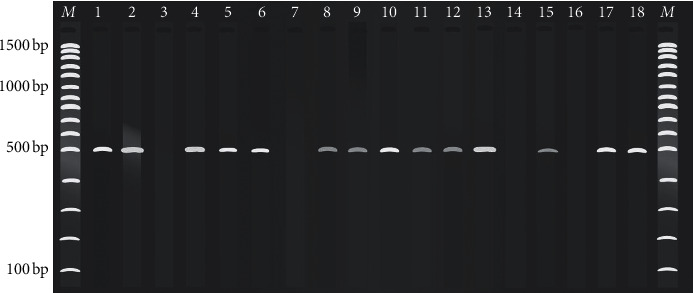
A gel image showing 500 bp marker of ITS1-5.8S-ITS2 region of *A. flavus* isolates from groundnuts amplified using primers FLA1/FLA2. Lanes 1–18 represent isolates, while *M* is a 100 bp DNA molecular ladder.

A single band of approximately 500 bp characteristic of *A. flavus* amplified ITS was observed on 14 isolates out of the 18 suspected isolates (Figure 4). This indicates that these isolates matched the DNA of either *A. flavus or A. oryzae*. There were no bands formed on isolates 3, 7, 14, and 16 showing that these isolates did not contain DNA from *A. flavus* or *A*. *oryzae*.

#### 3.3.1. Aflatoxin Gene Profile

The isolates varied greatly in their aflatoxin gene profiles (Table 2). At least one gene per isolates produced a detectable signal. Isolates 5, 11, 12, 13, and 15 had all the genes expressed, isolates 6 and 10 had 4 of their genes expressed, isolates 1, 4, and 18 had 3 of their genes expressed, and isolate 17 had only 1 gene expressed (Table 2). The regulatory gene *aflR* was expressed by all the isolates (100% expression), while the other regulatory gene *aflS* was expressed by 44% of the isolates. The structural genes *aflQ*, *aflP,* and *aflD* were expressed by 56%, 50%, and 44% of the isolates, respectively (Table 2).

#### 3.3.2. Sequence Analysis

**Figure 5:**
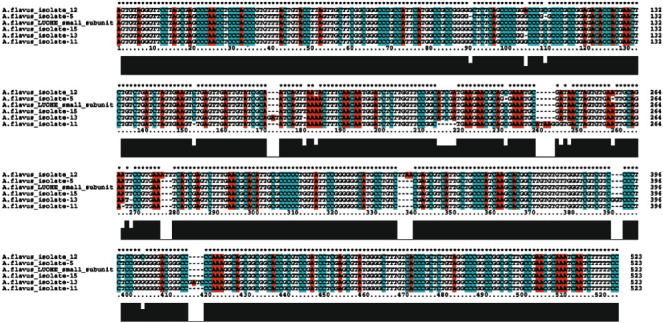
Nucleotide sequence alignment of *A. flavus* isolates with the isolate LUOHE small subunits ITS1-5.8S-ITS regions (GenBank accession no. MT645222.1). *∗∗∗*Indicates the conserved bases among the nucleotide with gaps indicating the unconserved regions.

The sequence data was obtained from the 5 isolates that had all their aflatoxin genes expressed: isolate 5, isolate 11, isolate 12, isolate 13, and isolate 15. The sequences were blasted on the NCBI database and aligned with the *A. flavus* isolate LUOHE accession number MT645222.1 that had the highest similarity to the isolates. Isolate 12 had 98.4% similarity, isolate 5 had 99.2% similarity, isolate 11 had 97.5% similarity, isolate 13 had 97.5%, and isolate 15 had 100% similarity to the *A. flavus* isolate LUOHE (Figure 5). The sequences were submitted to the NCBI GenBank and the accession numbers allocated as follows: LC567154, LC567155, LC567156, LC567157, and LC567158 for isolates 12, 5, 11, 13, and 15 respectively.

## 4. Discussion

The aflatoxins production is a major source of food security threat. Majority of households in Kenya are exposed to acute or chronic aflatoxicosis depending on ingestion levels, susceptibility, age, gender, and duration of exposure to aflatoxin [36]. Studies conducted by [9] indicate that there is as high as 2277.1 ppb aflatoxin contamination level in groundnuts from traders in Western and Nairobi provinces of Kenya. Acute aflatoxicosis in Kenya was reported in 2005 and 2004 growing season affecting 317 individuals of which 125 died [36]. Additionally, most infants are exposed to high level of aflatoxins in maize and sorghum-based diets and to aflatoxin MF1 (AFMI) through animal milk and breast milk [37]. The AFM1 in urine, stunted growth, and low weight gain in infants in Makueni County of Kenya indicates the high level of chronic aflatoxicosis [37]. This indicates that aflatoxin infection starts at early stages of development and progressed into adult hood. This emphasized the need to be more vigilant on aflatoxin causes, mitigation factors, and control.

Identification of the causative agent is a critical step in disease control. Morphological characterization is the commonly adopted method for fungal isolation and characterization. It employs the use of culture media to aid the growth and establishment of the fungus for observation. Growth media such as malt extract agar (MEA), sabouraud dextrose agar (SDA), rose bengal chloramphenicol agar (RBCA), and czapek dox agar (CZA) [16] and potato dextrose agar (PDA) [16, 33] have previously been used. These media can provide adequate requirements for fungus colony establishment that allows the development of the macroscopic and microscopic features suitable for assessment [16, 33]. This study employed the use of PDA which provided adequate growth and sporulation of the fungus allowing satisfactory evaluation.

Descriptive taxonomic keys were used as the initial fungal isolation criteria aiding the selection of presumptive *A. flavus* isolates. The colony growth started as a white mycelium that grew radially to cover the entire surface of the media. When sporulation began, a yellowish green or dark green colour of the conidia replaced the white colony colour from the centre outwards, eventually covering the entire surface. This was consistent with Thathana et al. [16] who found a white colour of the mycelia which produced an olive or dark green conidia delineated with a white ring. The colonies observed in this study had velvety to woolly texture often with floccose centre and cream colour on the reverse. Similar characteristics were reported by Thathana et al. [16], Bastianelli and Le Bas [38], and Odhiambo et al. [39]. The isolates were confirmed to belong to *Aspergillus* genera by presence of conidiophores, a key feature of the *Aspergillus* spp. [39]. However, the conidiophores observed in this study had a rough texture (Figure 2(c)) and were unbranched.

The vesicles were found to be subglobose to globose and varied in diameter with a biseriate sterigmata or philiades that radiated from all sides. This agreed with the preceding research results of Thathana et al. [16], Rodrigues et al. [19], and Diba et al. [33]. The metulae were borne on the vesicles in which the philiades arose while the globose, thin walled, and slightly roughened conidia that differed in sizes were borne on the tip of the philiades. These features were typical of the descriptive taxonomic keys provided by Klich [17] and in harmony with *A. flavus* characteristics previously documented by Thathana et al. [16], Rodrigues et al. [19], Diba et al. [33], and Odhiambo et al. [39]. Although morphology-based taxonomical keys are used as the initial isolation and identification criteria, they have various shortcomings and may not achieve accurate identification of the target fungal isolates [19, 20]. Therefore, further distinction among the groups/sections needs a comprehensive scrutiny using molecular means such as PCR based methods, gene expression, and sequence analysis.

Using the FLA1/FLA2 primers, the ITS1-5.8S-ITS2 region of the ribosomal DNA was amplified in 14 out of the 18 isolates that were presumptive representatives of the *A. flavus* confirming that they were positive for either *A. flavus* or *A. oryzae*. This was in agreement with the protocol developed by González-Salgado et al. [24]. The primers are designed to align to a more variable region of the ITS and they specifically amplify the *A. flavus* target sequence. The other four isolates formed no bands suggesting that the genomic DNA was from other genera or members within the *Aspergilli* section Flavi that had high morphological resemblance to the *A. flavus* or *A. oryzae*. Although *A. flavus* can be differentiated from other *Aspergilli* by morphology and amplification of the ITS sequence of the rDNA, it is very difficult to distinguish it from *A. oryzae* as morphological characteristics of these two fungi are similar [24]. To overcome the problem, expression of aflatoxin biosynthetic genes at mRNA level can be employed since these genes are ascertained to be silent in *A. oryzae* [27].

Scherm et al. [40] reported strong connection between *aflD, aflP,* and *aflQ* gene expression to aflatoxin production capacity. Furthermore, targeting *aflD* and *aflP* as documented by Peterson [41] specifically detects aflatoxin producing strains and excludes those that have a common sterigmatocystin pathway. The regulatory gene *aflR* involvement in the aflatoxin production has been ascertained and thus its action provides adequate information on the aflatoxin production capacity as compared to the activity of *aflS* which is less characterized [30]. The *aflR* gene is considered to increase the precision of discrimination and its expression was shown to have a good correlation with the aflatoxin synthesis in *A. flavus* strains [40, 42, 43]. Therefore, it is an accepted paradigm that the activation of this regulatory gene is necessary for the action of the structural genes.

In this study, one of the regulatory genes, *aflR,* was activated in all isolates unlike *aflS* which was expressed by only 44% of the isolates. This upholds the report of Chang [30] that the former gene provides more reliable information on the aflatoxin production capacity than the latter. Isolates 2, 9, and 18 had only one structural gene expressed out of the three that were targeted. The lack of the expression of some genes by some isolates even after expression of some or both of the regulatory genes could mean that they were weak aflatoxin producers and that the gene expression could not be detected. On the other hand, isolates 8 and 17 had none of the targeted structural genes expressed and may be considered atoxigenic strains of *A. flavus* or *A. oryzae*. These isolates were discarded since they were doubtful and it is better to have a false negative than false positive in the identification process. Isolates 5, 11, 12, 13, and 15 had all their genes expressed and thus they were considered to have the highest aflatoxin producing capacity.

The specific amplicons derived from isolate 5, 11, 12, 13, and 15 from the PCR were sequenced. The multiple sequence analysis of these isolates against *A. flavus* isolate LUOHE ITS-5.8S-ITS2 as a reference was done with Clustal X 2.1. It revealed a complete sequence similarity to isolate 15 but had slight changes within the gene sequences of the other isolates (Figure 5). As expected, when the genomic DNA is from the *A. flavus* or *A. oryzae*, the 5′ and 3′ end of the gene were conserved in all the sequences. The slight differences within the region did not change the amino acids produced by these isolates.

## 5. Conclusion

Identification of fungi within the *Aspergillus* genera is a complicated venture that requires an integrated approach to attain a reliable identification and characterization of isolates capable of synthesizing aflatoxins. This study successfully cultured and isolated 5 toxigenic isolates of *A. flavus* and submitted them to the NCBI GenBank where they were allocated accession numbers LC567154, LC567155, LC567156, LC567157, and LC567158. Isolates 3, 7, 14, and 16 never formed a band and were considered not to be *A. flavus*. Isolates 1, 2, 4, 6, 8, 9, 10, 17, and 18 are *A. flavus* isolates but their capacity to produce aflatoxin was doubtful and thus were considered atoxigenic. The sensitivity of the methods used in the isolation led to the lower number of the isolates obtained. This may lead to underestimation of the risks imposed by the *A. flavus* in the sampled region but the detection of aflatoxigenic *A. flavus* by this study augmented the risk of aflatoxin contamination. Accurate identification of aflatoxigenic fungi is paramount to develop a mitigation measures against fungal infections and mycotoxin production. This study therefore recommends employment of similar fungal identification approach in continual random sampling and analysis of suspect food products for possible mycotoxins contamination.

## Figures and Tables

**Table 1:** RT-PCR targeted genes and primer sequences with their expected amplicon size.

Gene	Primer pair	Primer sequence (5′ ⟶ 3′)	Amplicon size (bp)
*aflR*	Forward	CCGTCAGACAGCCACTGGACACGG	300
	Reverse	TGACCCACCTCTTCCCCCACG	

*aflS*	Forward	GAACGCTGATTGCCAATGCC	1256
	Reverse	CGGTCAGGATGTTACTAAGC	

*aflD*	Forward	ACCGCTACGCCGGCACTCTCGGAC	400
	Reverse	GTTGGCCGCCAGCTCTGACACTC	

*aflP*	Forward	GTGGACGGACCTAGTCCGACATCC	624
	Reverse	GTCGGCGCCACGCACTGGGTTGGG	

*aflQ*	Forward	TTAAGGCAGCGGAATACAAG	599
	Reverse	GACGCCCAAAGCCGAACACAAA	

*tub1*	Forward	GCTTTCTGGCAAACCATCTC	1198
	Reverse	GGTCGTTCATGTTGCTCTCA	

**Table 2:** Gene expression in 14 *A. flavus* isolates indicating their aflatoxin capacity.

Isolate	Regulatory genes		Structural genes		
	*aflR*	*aflS*	*aflD*	*aflP*	*aflQ*
1	+	−	+	+	−
2	+	−	−	−	+
4	+	−	+	−	+
5	+	+	+	+	+
6	+	−	+	+	+
8	+	+	−	−	−
9	+	−	−	+	−
10	+	+	−	+	+
11	+	+	+	+	+
12	+	+	+	+	+
13	+	+	+	+	+
15	+	+	+	+	+
17	+	−	−	−	−
18	+	+	−	−	+

Key: +, means expressed; −, means not expressed.

## Data Availability

Most of the data used to support the findings of this study are included in the article. Additional data are available from the corresponding author upon request.
